# Transcriptional profiling of the spleen in progressive visceral leishmaniasis reveals mixed expression of type 1 and type 2 cytokine-responsive genes

**DOI:** 10.1186/s12865-014-0038-z

**Published:** 2014-11-26

**Authors:** Claudia M Espitia, Omar A Saldarriaga, Bruno L Travi, E Yaneth Osorio, Alvaro Hernandez, Mark Band, Mandakini J Patel, Audrie A Medina, Michael Cappello, Andrew Pekosz, Peter C Melby

**Affiliations:** Department of Medicine, Cancer Therapy and Research Center, Institute for Drug Development, The University of Texas Health Science Center, San Antonio, Texas USA; Department of Internal Medicine, University of Texas Medical Branch, Galveston, Texas USA; Department of Microbiology and Immunology, University of Texas Medical Branch, Galveston, Texas USA; Center for Tropical Diseases and Institute for Human Infection and Immunity, University of Texas Medical Branch, Galveston, Texas USA; Roy J. Carver Biotechnology Center, University of Illinois, Urbana, Illinois USA; Department of Pediatrics, Yale School of Medicine, New Haven, Connecticut USA; Department of Molecular Microbiology and Immunology, Johns Hopkins University, Baltimore, Maryland USA; Department of Pathology, University of Texas Medical Branch, Galveston, Texas USA

**Keywords:** Hamster, *Leishmania donovani*, Visceral leishmaniasis, Transcriptional profiling, Microarray, Interferon-gamma, Interleukin-4

## Abstract

**Background:**

The Syrian golden hamster (*Mesocricetus aureus*) has been used as a model to study infections caused by a number of human pathogens. Studies of immunopathogenesis in hamster infection models are challenging because of the limited availability of reagents needed to define cellular and molecular determinants.

**Results:**

We sequenced a hamster cDNA library and developed a first-generation custom cDNA microarray that included 5131 unique cDNAs enriched for immune response genes. We used this microarray to interrogate the hamster spleen response to *Leishmania donovani*, an intracellular protozoan that causes visceral leishmaniasis. The hamster model of visceral leishmaniasis is of particular interest because it recapitulates clinical and immunopathological features of human disease, including cachexia, massive splenomegaly, pancytopenia, immunosuppression, and ultimately death. In the microarray a differentially expressed transcript was identified as having at least a 2-fold change in expression between uninfected and infected groups and a False Discovery Rate of <5%. Following a relatively silent early phase of infection (at 7 and 14 days post-infection only 8 and 24 genes, respectively, were differentially expressed), there was dramatic upregulation of inflammatory and immune-related genes in the spleen (708 differentially expressed genes were evident at 28 days post-infection). The differentially expressed transcripts included genes involved in inflammation, immunity, and immune cell trafficking. Of particular interest there was concomitant upregulation of the IFN-γ and interleukin (IL)-4 signaling pathways, with increased expression of a battery of IFN-γ- and IL-4-responsive genes. The latter included genes characteristic of alternatively activated macrophages.

**Conclusions:**

Transcriptional profiling was accomplished in the Syrian golden hamster, for which a fully annotated genome is not available. In the hamster model of visceral leishmaniasis, a robust and functional IFN-γ response did not restrain parasite load and progression of disease. This supports the accumulating evidence that macrophages are ineffectively activated to kill the parasite. The concomitant expression of IL-4/IL-13 and their downstream target genes, some of which were characteristic of alternative macrophage activation, are likely to contribute to this. Further dissection of mechanisms that lead to polarization of macrophages toward a permissive state is needed to fully understand the pathogenesis of visceral leishmaniasis.

**Electronic supplementary material:**

The online version of this article (doi:10.1186/s12865-014-0038-z) contains supplementary material, which is available to authorized users.

## Background

The Syrian golden hamster (Mesocricetus aureus) is susceptible to, and has been used as a model for, a number of human pathogens, including *Leishman*ia *(Viannia)* spp. [1–4], *L. donovani* [5,6], *Trypanosoma cruzi* [7], *Entamoeba histolytica* [8], *Leptospira* and *Treponema* [9,10], hantavirus [11], Eastern equine encephalitis virus [12], Yellow Fever virus [13,14], West Nile virus [15], Nipah virus [16], and hookworm [17]. In some cases, hamster models of infection provide a unique opportunity to determine mechanisms of disease and immunity because the human infection is more closely mimicked in this animal than in other animal models. Studies of immunopathogenesis in hamster infection models are challenging, however, because of the limited availability of reagents needed to define cellular and molecular determinants. For the most part, antibodies directed against mouse and rat proteins do not cross-react with the hamster orthologs, and the lack of a fully annotated genome sequence limits broad interrogation of the genome and transcriptome.

The leishmaniases are a diverse group of diseases caused by intracellular protozoan parasites of the genus *Leishmania*. Visceral leishmaniasis (VL) is one of the “Neglected Tropical Diseases” that impacts the most resource-poor regions of the world. Active VL, caused by *L. donovani*, is characterized by a progressive increase in visceral parasite burden, cachexia, massive splenomegaly, pancytopenia and ultimately death. There are significant gaps in our understanding of the molecular and cellular determinants underlying the pathogenesis of VL. The Syrian hamster (*Mesocricetus auratus*) affords a unique opportunity to address these gaps because the clinicopathological features of VL in the hamster closely mimic active human disease. In recent reports [5,18] we demonstrated that despite mounting a vigorous Type 1 cellular immune response, an immunological event that is associated with control of infection in mice, the hamster develops a progressive, lethal disease. This paradoxical finding was reminiscent of the findings in humans [19,20]. Furthermore, we found that the inability of *L. donovani* infected hamsters to control parasite replication was related to ineffective IFN-γ-mediated classical macrophage activation, evident by reduced expression of inducible nitric oxide synthase (NOS2) and production of nitric oxide (NO), which is the primary mechanism by which mice control *Leishmania* infection [5,18]. We found that parasitized macrophages were not deactivated but showed a M2 (“alternatively activated”) phenotype where the expression of host arginase 1 (arg1) dominated at the site of infection [21,22]. Although it is a well-established paradigm that M2 macrophages are driven by Th2 cytokines, we discovered that *L. donovani* infection of macrophages and fibroblasts induced the expression of arg1 through an IL-4-independent, but STAT6 dependent, mechanism [21,22]. Furthermore, the activation of STAT6 and expression of arg1 enhanced intracellular parasite replication [21,22]. To better define the splenic environment that leads to a failure of host defense, we investigated the splenic response to *L. donovani* infection in the hamster model of progressive VL by use of a custom cDNA microarray. We show that following a relatively silent early phase of infection there is dramatic upregulation of inflammatory and immune-related genes in the spleen that is coincident with the exponential increase in parasite replication [21,22]. The gene expression profiling identified a mixed cytokine response of IFN-γ, IL-4 and IL-10 with corresponding expression of a large number of cytokine-responsive genes in VL.

## Results and discussion

### Hamster cDNA sequence assembly, characterization, and annotation

As noted above there are a number of experimental infection models in Syrian hamsters that are relevant to human disease [1–17]. However, there is limited availability of molecular tools for studies of disease pathogenesis in this model. A draft genome of *Mesocricetus auratus* determined via genome shotgun sequencing has been reported (NCBI Accession APMT01000001), but it was incompletely annotated at the time when the data presented here were being analyzed. As an initial approach to address this obstacle we sequenced a Syrian hamster cDNA library constructed from a pool of mRNA that had been isolated from 1) spleen, LN cells, and peritoneal macrophages exposed to various stimuli, and 2) normal tissue or tissue harvested from hamsters infected in vivo with several different pathogens. We chose to use cells and tissues that had been exposed to a broad range of stimuli and pathogens (bacteria, viruses, protozoa, and helminths) in order to enrich for a diverse set of mRNAs involved in immune responses. From the cDNA library 10,000 independent clones were sequenced to obtain 5085 unique expressed sequence tags (EST). Datasets representing all sequences were assembled into contigs of overlapping sequences using Phred (for accurate base-calling from DNA sequence traces) and Phrap (for fast and accurate DNA sequence assembly), and were compared to the non-redundant nucleotide database using the BLAST algorithm [23]. Sequences that had a significant match with a mouse, rat, or human sequence were considered Syrian hamster orthologs of the closest match. The breakdown of closest match by non-hamster species is shown in Additional file 1: Table S1. Hamster cDNAs had the highest level of homology with mouse (49.6%) and rat (27.7%) sequences; 12.9% did not have a significant match to the GenBank database. Only 4.5% of sequences showed the highest homology to human or non-human primate DNA and 3.7% of sequences matched to non-mammalian species and were likely of pathogen origin since RNA from the protozoa, helminthes, or viruses in the infected tissue would have been included in the RNA used to construct the library (see Additional file 1: Table S1).

### Analysis of splenic gene expression by microarray

The immunopathogenic mechanisms that contribute to visceral leishmaniasis (VL) are not clearly understood. In a model of progressive VL [5,6,18,21,22] we investigated splenic gene expression over the course of infection using a first generation cDNA custom microarray. 5085 unique ESTs from the cDNA library, and an additional 46 cDNAs that we had cloned independently (references [5,6,18,22,24], and Melby, unpublished) were amplified and spotted in duplicate onto a microarray making a total of 5131 unique cDNAs plus replicates of several housekeeping genes. We used the microarray to define the expression of splenic mRNAs in response to visceral *L. donovani* infection at 7, 14, and 28 days post-infection.

**Figure 1:**
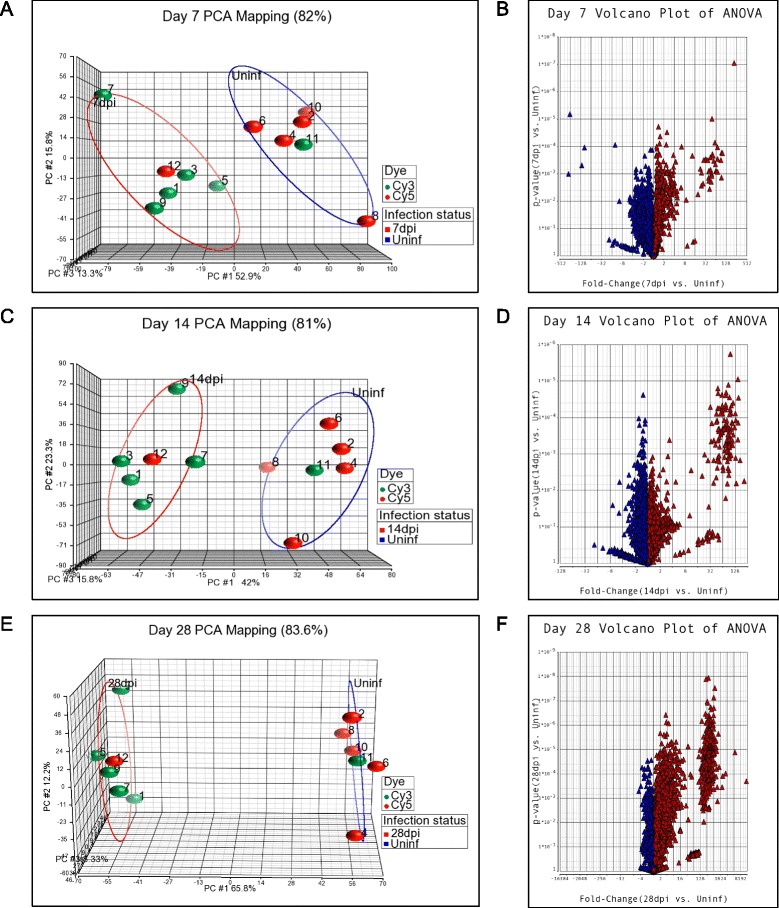
**Principle Component Analysis and Volcano Plots of transcripts expressed in the spleens of uninfected or infected hamsters. A**, **C**, **E**. Principle Component Analysis of uninfected and infected spleen tissue at 7 days **(A)**, 14 days **(C)**, and 28 days **(E)** post-infection. The groups of uninfected samples are circled in blue and infected samples are circled in red. The Cy3 and Cy5 dyes are represented by green and red symbols, respectively. Dye-swap arrays (files 11 and 12 for each time point) from pooled samples clustered appropriately within the corresponding infection status. **B**, **D**, **F**. Volcano plots showing the mean fold-change and p-value for the comparisons of uninfected and infected spleen tissue at 7 days **(B)**, 14 days **(D)** and 28 days **(F)** post-infection. Red triangles show a fold-change >1 and blue triangles show a fold-change <1.

Using Partek GS, a Principal Component Analysis (PCA) was performed to identify major effects influencing the expression values at each time point [25]. This is an exploratory global data analysis of the genome and not an analysis of any gene in particular. It was used for quality control of the data on all the samples (Figure 1). In general, samples that map closely are similar across the whole genome, whereas samples that map distantly are dissimilar across the whole genome [26]. A distinct clustering between uninfected and infected groups was clearly identified, showing that the infection status, and not a batch effect due to Cy5 or Cy3 labelling or the file (representing a couple of infected and uninfected hamsters/spleen tissues) had the largest effect on the data at all time points. Also no outliers were identified; dye-swap arrays (files 11 and 12 for each time point) from pooled samples clustered within the correspondent infection status (Figure 1). Thus, infection status had the major impact on differential gene expression in the spleens.

After curation of the differentially expressed EST, the lists were reduced to identifiable differentially expressed hamster genes. We demonstrated previously that in the hamster model of VL there is an initial period of silent infection, with little activation of the immune response, followed by a sharp increase in spleen size, number of macrophages, and splenic parasite burden [5,21,22]. The gene expression data presented here corroborated this previous observation at a much broader transcriptomic level (Table 1), but it must be recognized that minor changes in gene expression may not be detected by microarray. When ESTs that had at least a 2-fold change in the mean expression between the uninfected and infected groups (n = 5 per group) and a False Discovery Rate (FDR) of <5% were considered to be differentially expressed, 8 and 24 genes were differentially expressed at 7 and 14 days post-infection, respectively, but 708 genes were differentially expressed at 28 days post-infection (Table 1 and Additional file 2: Table S2, Additional file 3: Table S3 and Additional file 4: Table S4). At this latter time point we demonstrated previously that there was a dramatic increase in parasite burden and accumulation of cells, especially macrophages, in the spleen [21,22]. When at least a 2-fold change and a *p* value <0.05 was used as the threshold for significance the number of differentially expressed genes was higher. In the analysis of the data presented here we used a >2-fold change and FDR <5% to define the set of differentially expressed genes.

**Table 1:** **Differentially expressed EST in the hamster spleen infected for 7, 14, and 28 days compared to uninfected controls**

**Days post-infection**	**Number of differentially expressed EST (Infected vs Uninfected)**	
	2FC and FDR <5%*	**2FC and p < 0.05**
7	8	313
14	24	158
28	708	1476

*FC = fold change; *FDR* = false discovery rate.

As shown in the volcano plots in Figure 1, most genes in the infected groups were expressed at less than a 2.0-fold increase or decrease over the age-matched uninfected control group. However, at each of the time points there was cluster of highly upregulated mRNAs, which increased over the course of infection such that at 28-days post-infection there were 165 mRNAs with a fold-increase of >20 (Figure 1; Additional file 5: Table S5). Only a small number mRNAs (n = 27) were down-regulated at 28-days post-infection. This is contrary to the previously held view, derived from microarray data from *in vitro* infected mouse [27,28] and human [29] macrophages, that *Leishmania* infection had a broadly silent or suppressive rather than stimulatory effect on host transcription. However, these studies would not have included the multiple different cell populations and a myriad of inflammatory signals present in the whole infected spleen tissue. It should also be noted that the cDNA library from which the microarray was created included RNA isolated from *Leishmania*-infected hamsters so it is likely that the microarray was enriched for genes that were upregulated in that infection model. The number of down-regulated genes was therefore probably underestimated. Comparable transcriptome profiling has not been reported for the spleens of *L. donovani* infected mice or humans.

As noted earlier, *Leishmania* DNA may have been included in the microarray and/or *Leishmania* RNA could have cross-hybridized with an orthologous hamster EST. To minimize the possibility that *Leishmania* orthologs could confound the interpretation of the data, differentially expressed genes were ignored if the EST was obviously derived from a pathogen (including *Leishmania*). The possibility of a *Leishmania* RNA cross-hybridizing to the hamster ortholog (giving a false impression that the hamster transcript was upregulated) cannot be excluded. For some ESTs assigning a gene symbol was challenging. A case in point is Bbs1, in which there is a *Leishmania* ortholog of the mammalian gene [30]. This EST was identified as the most highly upregulated gene in the 28-day infected hamster spleen (Additional file 4: Table S4). It showed homology to the 3′ end of the mouse Bbs1 sequence (therefore was labeled as Bbs1), but did not have sequence homology to the incompletely annotated hamster (*Mesocricetus auratus*) genome or to the published *Leishmania* sequence. Using qRT-PCR and specific primers there was no increase in the transcript of the published hamster Bbs1 but a substantial increase in the *Leishmania* Bbs1 ortholog (data not shown). Therefore, the *Leishmania* Bbs1 ortholog was highly expressed in the spleen during infection, the EST labeled as Bbs1 based on homology to the mouse sequence was upregulated, but this differentially expressed gene was not identical to the published hamster Bbs1 sequence. Other similar examples may exist, but these would primarily concern housekeeping/metabolic genes so would not affect the interpretation of the immunological findings of this study.

**Figure 2:**
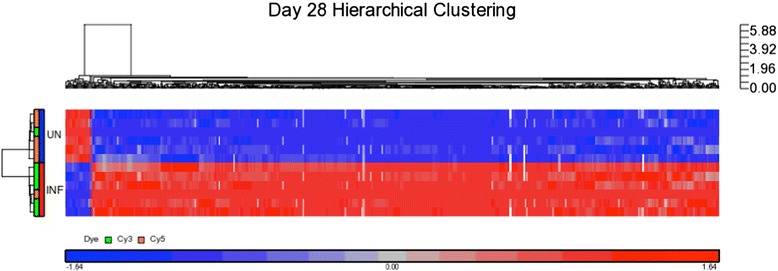
**Hierarchical Clustering of differentially expressed transcripts in the spleens of hamsters infected with** ***L. donovani*** **for 28 days.** Unsupervised hierarchical clustering of differentially expressed genes (>2-fold change and FDR <5%) in the spleens of uninfected (UN) and 28-day infected (INF) hamsters. The heatmap is color-coded using red for up-regulated genes and blue for down-regulated genes.

Hierarchical clustering using Euclidean distance and average linkage was applied to the differentially expressed genes at 28 days post-infection. The Heatmap and dendrogram of the samples showed a pattern consistent with the PCA, where arrays grouped under two major categories regardless of the labeling dye (Figure 2). The clustering of genes based on the similarity of their expression patterns suggests, but does not prove, that they have a similar biological function [31]. In general, all genes clustered into two expression groups, infected and uninfected. Because of the high number (708) of differentially expressed genes at 28-day infection, a gene ontology (GO)-enrichment analysis [32] using Partek GS was made to better understand their putative biological function (Table 2). High enrichment scores were detected for a number of biological functions that involved immune and inflammatory responses. This was not unexpected, since the cDNA library was designed to be enriched for immune-related genes. Identification of the genes included in the immune response and inflammatory response functions and their fold-increase is shown in Table 3.

**Table 2:** **Classification of differentially expressed genes by gene ontology** ^**1**^ **in the hamster spleen after 28 days of infection**

**Function**	**Type**	**Enrichment Score** ^**2**^	**Adjusted** ***p*** **-value** ^**3**^
Extracellular space	Cellular component	33.93	1.84E-15
Immune response	Biological process	23.35	7.22E-11
Inflammatory response	Biological process	22.44	1.80E-10
Extracellular region	Cellular component	21.32	5.52E-10
Chemokine activity	Molecular function	20.97	7.78E-10
Platelet degranulation	Biological process	20.44	1.33E-09
Chemotaxis	Biological process	19.46	3.52E-09
Blood coagulation	Biological process	19.30	4.14E-09

^1^Gene ontology (GO)-enrichment analysis using Partek GS.

^2^Ratio of the number of genes in the gene set to the expected number in the category based on the human database.

^3^p value adjusted by the multiple test adjustment.

**Table 3:** **Genes included in the “Immune Response” and “Inflammatory Response” Gene Ontology functions from differentially expressed genes after 28 days of infection**

**Immune response**			**Inflammatory response**		
**Gene symbol**	**Fold-change**	***p*** **-value**	**Gene symbol**	**Fold-change**	***p*** **-value**
*Ifng*	1748.95	9.71E-05	*Ccl11*	1212.26	0.00035
*Ccl11*	1212.26	0.00035	*Ccl20*	749.90	0.00133
*Ccl20*	749.90	0.00134	*Cxcl1*	488.25	4.04E-05
*Igha1*	653.51	8.07E-06	*Reg3g*	348.91	6.26E-05
*Cxcl5*	653.22	1.28E-04	*Chst4*	197.33	3.52E-05
*Csf3*	509.58	8.54E-06	*Cdo1*	45.39	0.0001
*Cxcl1*	488.25	4.04E-05	*Cxcl9*	35.64	7.46E-06
*Azgp1*	234.93	2.52E-05	*Ccl17*	16.36	0.020
*Chst4*	197.33	3.52E-05	*Ccr5*	13.75	3.60E-05
*Cxcl9*	35.64	7.46E-06	*Cxcl10*	12.16	2.25E-05
*Ccl17*	16.36	0.020	*Fpr2*	10.12	0.00073
*Il21*	14.64	6.74E-04	*Il1rn*	9.50	0.00026
*Ccr5*	13.75	3.60E-05	*Hrh1*	8.74	0.005
*Cxcl10*	12.16	2.25E-05	*Aoah*	6.33	0.00011
*Gbp2*	9.86	1.45E-05	*Kng1*	5.25	0.012
*Cst7*	9.53	0.00038	*C5*	5.17	0.022
*Il1rn*	9.50	0.00026	*Cybb*	4.97	0.00011
*IL4*	9.18	2.62E-05	*IL10*	4.57	0.00092
*IL2Ra*	8.15	0.0009	*Mif*	4.55	0.0117
*Cd8a*	6.73	0.0185	*IL6*	4.50	0.00059
*Oasl*	6.12	1.12E-04	*Ccl22*	3.68	0.00092
*IL2*	4.75	0.0122	*Cxcl2*	3.44	0.00723
*Gzma*	4.28	4.24E-04	*Cxcl3*	3.38	0.00965
*Igsf6*	4.15	0.0121	*Lipa*	3.02	8.24E-05
*Il12a*	4.14	0.0185	*C3*	2.96	2.79E-05
*Ctss*	3.88	0.0021	*Slc11a1*	2.72	1.04E-05
*Igha1*	3.79	4.57E-05	*Tirap*	2.28	0.00035
*Tlr2*	3.73	0.0018	*Nlrp3*	2.07	0.0166
*Ccl22*	3.68	0.00092			
*Ctss*	3.52	0.0134			
*CD86*	3.45	0.00068			
*Cxcl2*	3.44	0.0072			
*Cxcl3*	3.38	0.0096			
*Col4a3bp*	3.26	0.00036			
*C3*	2.96	2.79E-05			
*Map4k2*	2.65	0.00011			
*Cmklr1*	2.62	4.95E-05			
*Hfe*	2.51	0.00168			
*Gem*	2.50	0.00169			
*Osm*	2.39	0.0034			
*Pnp*	2.17	0.0031			
*Ikbke*	2.06	0.00073			
*Map4k2*	−2.07	0.0045			
*Cxcl12*	−2.72	0.00015			
*Tinagl1*	−2.88	0.0065			
*Ppbp*	−3.29	5.52E-05			

Analysis of the differentially expressed genes by the “WEB-based GEne SeT AnaLysis Toolkit” [33,34] and WikiPathways [35] revealed a number of pathways that were significantly upregulated by infection (Table 4). These analyses were no doubt limited by the incomplete representation of the hamster transcriptome in the custom microarray. Nevertheless, the transcripts that were differentially regulated at day-28 p.i. represented a broad array of genes involved in inflammation, immunity, immune cell trafficking, and metabolism. Of particular interest were the pathways involved in T cell subset polarization and effector function. The Enrichment Ratio (ER; ratio of differentially expressed genes in the gene set to the expected number in the pathway published for mice) was significantly increased for the Type II interferon (IFN-γ) (ER = 17.77; p = 1.18e-11) and IL-4 signaling pathways (ER = 12.14, 6.89e-07) in the infected animals. Because the custom microarray did not likely include all members of a pathway the numerator of the ER is probably artificially low. The denominator is set independent of the microarray (based on publications related to the pathway in mice), so the calculated Enrichment Ratio is likely to be an underestimate of the true significance.

**Table 4:** **Signaling pathways significantly upregulated at 28 days post-infection**

**Pathway Name**	**Enrichment ratio** ^**1**^	**Adjusted** ***p*** **value** ^**2**^
Amino Acid Metabolism	16.26	7.79e-15
Complement and Coagulation Cascades	21.56	1.66e-12
Adipogenesis	12.17	1.18e-11
Type II interferon signaling (IFN-γ)	17.77	1.18e-11
Chemokine signaling pathway	9.25	1.46e-10
Blood clotting cascade	39.54	2.74e-10
Toll-like receptor signaling pathway	11.04	1.69e-08
Cytokines and inflammatory Response (BioCarta)	12.47	6.10e-07
IL-4 signaling pathway	12.14	6.89e-07
IL-2 Signaling pathway	9.90	3.38e-06

^1^Ratio of the number of genes in the gene set to the expected number in the category based on the human database.

^2^ *p* value adjusted by the multiple test adjustment.

**Figure 3:**
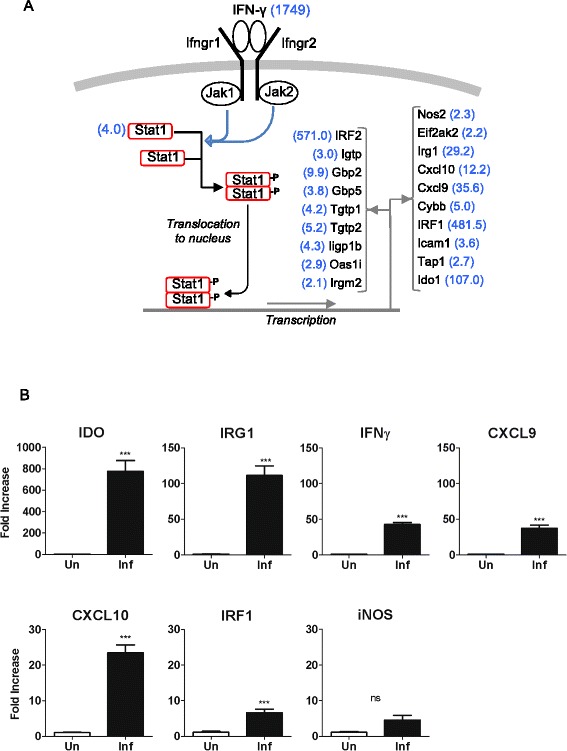
**Upregulation of Interferon-γ-inducible genes in the spleens of 28-day infected hamsters. (A)** Schematic representation of the IFN-**γ** pathway with notation of interferon-γ-inducible genes found by microarray to be upregulated in the spleens of hamsters at 28 days post-infection. The fold-increase of the mRNA in the infected tissue is shown in blue in parentheses. **(B)** Expression of interferon-γ-inducible genes in the spleens of 28-day infected hamsters determined by real time RT-PCR. Data are shown as the mean and standard error of the mean (error bars) of the fold-increase in infected compared to uninfected animals (n = 5 per group). ***p ≤ 0.001.

IFN-γ potently conveys antimicrobial properties to macrophages by inducing the transcription of a broad repertoire of genes [36]. In murine models of *Leishmania* infection, IFN-γ, produced primarily by CD4+ and CD8+ T cells, is critical to the control of infection (reviewed in [37]). Paradoxically, in the hamster model of progressive VL [5,18], as well as in humans with active VL [19,20,38], there are high levels of IFN-γ expression without effective control of infection. Indeed, in the microarray analysis, IFN-γ was the second most highly differentially expressed transcript (>1700-fold increase) at 28-days post-infection (Table 3; Additional file 5: Table S5), a time point in the course of infection when there is a massive increase in splenic parasite burden [21,22]. The reason(s) why IFN-γ (and its downstream targets) effectively controls the parasite burden over time in mice but not in hamsters is not fully understood. Furthermore, we found evidence of upregulation of a number of known interferon-responsive genes (Figure 3A). In independent experiments we verified the microarray results by confirming the increased expression of a subset of these IFN-induced transcripts (indoleamine dioxygenase-1 [*Ido1*], immune-responsive gene 1 [*Irg1*], CXC chemokine ligand 9 [*Cxcl9*], *Cxcl10*, and interferon regulatory factor-1 [*Irf1*]) in the spleens of 28-day infected hamsters by qRT-PCR (Figure 3B). Notably, and consistent with our previous work, we found minimal increase in inducible nitric oxide synthase 2 (*Nos2*) expression, which is the primary effector mechanism for macrophage-mediated killing of intracellular *Leishmania* in mice [39,40]. We previously identified sequences in the hamster NOS2 promoter that rendered it less responsive to classical activating stimuli [41]. However, the transcriptional profiling presented here revealed an additional mechanism by which Nos2 may be suppressed in macrophages. The high level of expression of the transcription factor IRF2 (Table 3; Figure 3A), which is synergistically induced by IFN-γ and IL-4 [42], may compete with IRF-1 to repress the transactivation of Nos2 and other IFN-inducible genes [42,43]. This possible mechanism of impaired Nos2 expression remains to be explored.

The potential roles of other IFN-γ-responsive genes in the defense against *Leishmania* are incompletely understood. The expression of indoleamine 2,3-dioxygenase-1 (*Ido1*) and tryptophan 2,3-dioxygenase (*Tdo*), which can limit pathogen replication by local depletion of tryptophan [44–46], was significantly increased in the spleens of infected animals (Additional file 4: Table S4; Figure 3A and 3B). However, consistent with our results, recent work determined that the expression of Ido1 may suppress both innate and adaptive anti-leishmanial immune responses [47,48] and Ido-1 is a marker of disease activity in human VL [49]. The expression of *Cxcl9* and *Cxcl10*, which signal through the Cxcr3 receptor, was greatly increased in the spleens of hamsters with VL (35- and 12-fold, respectively) (Table 3; Figure 3A and 3B; Additional file 4: Table S4) and they were elevated in the serum of patients with VL [50]. Their role in defense against VL is uncertain. In *L. major* infected mice Cxcr3 was required for resolution of infection through recruitment of effector T cells to the site of infection [51]. In *L. donovani* infected mice, however, Cxcr3 was not required for development of a Th1 response and control of hepatic infection [52]. A number of transcripts from the family of IFN-inducible GTPases (*Igtp*, *Iigp1b*, *Irgm2*, *Tgtp1*, *Tgtp2*, *Gbp2*, *Gbp5*) were increased in the spleen of hamsters with VL (Figure 3A; Additional file 4: Table S4). This family of proteins includes the immunity-related GTPases (p47 IRGs) and p65 guanylate binding proteins (p65 GBPs) that promote resistance against intracellular bacterial, viral and protozoal pathogens (reviewed in [53]). Targeted disruption of some, but not all, of these genes in mice enhanced susceptibility to dermatropic *Leishmania* strains [54] but the role of this family in *L. donovani* infection is unknown. Our data indicate that their expression does not preclude the progression of VL. Collectively, these data reinforce the notion that progressive VL is not driven by the absence of IFN-γ production or a global deficit in IFN-γ responsiveness, but that other factors lead to unresponsive macrophage effector function.

**Figure 4:**
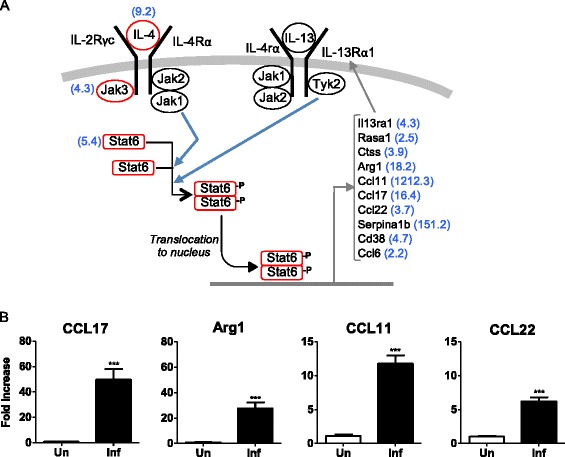
**Upregulation of IL-4/13-inducible genes in the spleens of 28-day infected hamsters. (A)** Schematic representation of the IL-4 and IL-13 signaling pathways with notation of IL-4/IL-13-responsive genes found by microarray to be upregulated in the spleens of hamsters at 28 days post-infection. The fold-increase of the gene in the infected tissue is shown in blue in parentheses. **(B)** Expression of IL-4/IL-13-responsive genes in the spleens of 28-day infected hamsters determined by real time RT-PCR. Data are shown as the mean and standard error of the mean (error bars) of the fold-increase in infected compared to uninfected animals (n = 5 per group). ***p ≤ 0.001.

The type 2 cytokines, interleukin (IL)-4 and IL-13, are likely to contribute to the pathogenesis of experimental VL. IL-4 expression was increased 9.2-fold in the spleens of 28-day infected compared to uninfected hamsters (Table 3; Figure 4A; Additional file 4: Table S4), confirming our previous findings [5,6,22]. IL-13 was increased 8.2-fold, however, it did not meet the FDR criteria for a differentially expressed gene. By quantitative RT-PCR we recently determined that its expression was increased in the spleen of hamsters with VL [22]. IL-4 and IL-13 signal through pathways that have considerable overlap: the IL-4 and IL-13 receptors share the IL-4Rα chain and activation of the STAT6 transcription factor is central to their canonical pathways (see Figure 4A). The action of IL-4, and to a lesser extent IL-13, plays a prominent role in the immunopathogenesis of murine *L. major* infection (reviewed in [55]). However, in mice infected with *L. donovani*, where parasite replication is ultimately controlled and the animals do not develop progressive disease, IL-4 and IL-13 do not contribute to host susceptibility [56,57]. In fact, IL-13 promotes the formation of hepatic granulomas and control of murine *L. donovani* infection [58]. The significance of IL-4 and IL-13 in human VL has not been fully addressed. IL-4 is increased in the spleens [59] and IL4 and IL-13 are increased in the plasma or serum [59–61] of patients with active VL. Our previous work in the hamster model of progressive VL identified STAT6-dependent, IL-4-amplifying M2-like macrophage polarization as a critical determinant of progressive disease [22]. The microarray data presented here revealed that a number of IL-4/IL-13-responsive genes were upregulated during infection (Figure 4A; Additional file 4: Table S4), including genes that are characteristic of alternatively activated (M2) macrophages. The increased expression of arginase 1, CC chemokine ligand 17 (*Ccl17*), *Ccl22*, and *Ccl11* was confirmed by real time RT-PCR (Figure 4B). An M2b-like phenotype of macrophages (which demonstrated increased expression of arg1, Mrc1, Clec7a, CCL17, and IL-10) was also reported in mice infected with *L. infantum*, a closely related visceralizing species [62].

## Conclusions

First generation microarray analysis of gene expression in the spleens of hamsters with progressive VL demonstrated a large number of highly upregulated genes. While the microarray provided only partial coverage of the hamster transcriptome, transcripts that were part of the IFN-γ and IL-4/IL-13 signaling pathways were prominently expressed. A robust and functional IFN-γ response is clearly evident by the expression of multiple downstream target genes, but this response does not restrain parasite replication. This finding supports the accumulating evidence that macrophages are ineffectively activated to kill the parasite in the spleens of progressive VL. The concomitant expression of IL-4/IL-13 and IL-10, with the increased expression of genes characteristic of M2 macrophage activation, are likely to contribute to this. The fundamental mechanism(s) through which the high level of IFN-γ expression is unable to suppress type 2 cytokine response and mediate classical macrophage activation remains a mystery. Further dissection of the regulatory mechanisms that lead to polarization of macrophages toward a permissive state is needed to fully understand the pathogenesis of VL.

## Methods

### Parasites

*Leishmania donovani* (MHOM/SD/001S-2D) promastigotes were cultured as described previously [63]. Hamsters were infected by intracardial injection of 10^6^ peanut agglutinin purified metacyclic promastigotes [63].

### Animals

Outbred Syrian hamsters were used for isolation of total RNA to construct a cDNA library. For gene expression studies, randomly selected, age-matched female inbred Chester Beatty hamsters were either uninfected or infected with 1 × 10^6^ metacyclic *Leishmania donovani* promastigotes by intracardial injection. Animals (n = 5 per uninfected and infected groups) were sacrificed at 7, 14 and 28 days post-infection and the spleens collected and frozen at −80°C in RNAlater (Ambion) until the RNA was isolated. Animals used in this study were handled according to local and federal regulations. The research protocols were approved by our Institutional Animal Care and Use Committee (Protocols 04051-41-05 at UTHSCSA and 1101004 at UTMB). The ARRIVE Guidelines [64] for reporting animal research were followed.

### Isolation of RNA for the hamster cDNA library

Multiple different hamster cell populations and tissues were exposed or not to several different noninfectious or infectious stimuli. Hamster cell and tissue sources of mRNA used in the construction of the cDNA library included: spleen cells: no stimulus, 100 μg/mL Concanavalin A, 100 μg/mL LPS; peritoneal macrophages: no stimulus, 100 μg/mL LPS; popliteal lymph node tissue from hamsters with cutaneous (foot) *Leishmania panamensis* infection; mesenteric lymph node tissue from hamsters with intestinal *Ancylostoma duodenale* infection; spleen tissue from normal animals; spleen tissue from animals with systemic *Leishmania donovani* infection; liver tissue from normal animals; liver tissue from animals with systemic *Leishmania donovani* infection; epithelial cells infected with influenza A virus, SARS-CoV, Andes virus or treated with hamster interferon-alpha. Total RNA was isolated using the RNAeasy (Qiagen) kit and pooled for the construction of the cDNA library.

### Construction of cDNA library

A subtractive directionally cloned cDNA library was constructed and sequenced at the Roy J. Carver Biotechnology Center, University of Illinois at Urbana-Champaign. The library was prepared as described by Bonaldo et al. [65] following method 4 with some modifications. Briefly, the poly(A) + mRNA was isolated from total RNA using the Oligotex Direct mRNA kit (Qiagen) and converted to double-stranded cDNA (dscDNA) using the Superscript® Double-Stranded cDNA Synthesis Kit and an anchor primer 5′-AACTGGAAGAATTCGCGGCCGCACCGATTTTTTTTTTTTTTTTTTV-3″ (V = A,C,G). The dscDNAs were size selected on a 1% agarose gel (>0.5 kb), ligated to EcoR1 adaptors (Promega) and digested with NotI (NEB). The dscDNAs were then directionally cloned into EcoR1-NotI digested pBluescript II SK + phagemid vector (Stratagene). The ligated dscDNAs were transformed into DH10B™cells (Invitrogen). The total number of white colony forming units (cfu) before amplification was 4 × 10^6^. Blue colonies (empty vectors) were less than 2%.

### Normalization/subtraction of the primary hamster cDNA library

Purified plasmid DNA from the primary library was converted to single-stranded circles and used as a template for PCR amplification using the T7 and T3 priming sites flanking the cloned cDNA inserts as previously described [65]. The purified PCR products, representing the entire cloned cDNA population, and PCR products from clones found to be abundant in the primary library were used as a driver for normalization. Hybridization between the single-stranded library (50 ng) and the PCR products (500 ng) was carried out for 44 hours at 30°C. Unhybridized single-stranded DNA circles were separated from hybridized DNA, rendered partially double-stranded and electroporated into DH10B™ cells to generate the normalized/substracted library. The total number of clones was 1.5 × 10^7^ cfu. Background of empty clones was less 2%.

### Sanger sequencing of cDNA inserts

Transformed colonies were picked and plated in 384 well plates in LB containing carbenicillin. Plasmids were extracted and sequenced on Applied Biosciences 3730xl sequencers. Base calling with quality score was carried out using Phred. Vector sequence was detected and trimmed using Cross-Match. Sequences were considered high quality with average Phred scores of 20 or above and a minimum of 200 bp after quality and vector trimming. 5085 raw Expressed sequence tags (EST) were generated from the library. These ESTs were further assembled into contigs. Sequence data were integrated in a Hamster ‘Expressed Sequence Tag Information Management and Annotation’ (ESTIMA) database [66].

### cDNA Oligonucleotide printing, microarray slide preparation and processing

cDNA microarray slides were printed based on EST/Contigs of candidate genes provided by the cDNA library sequence. PCR products for printing were synthesized directly off bacterial culture as template. The DNA amplicons were resuspended in printing buffer using the Beckman Biomek FX liquid handling robot. Printing (spotting) of slides, including slide preparation, and setting up the software, was done at the W. M. Keck Center for Comparative and Functional Genomics at the University of Illinois at Urbana-Champaign. Slides were printed robotically with a GeneMachines OmniGrid 100 microarray printer.

### cDNA synthesis, labeling and hybridization

Total RNA was extracted from spleen tissue from each uninfected and infected hamster using the RNeasy Midi kit (Qiagen, Foster City, CA). Each RNA sample was DNase treated with TURBO DNA-freeTM kit (Ambion®) and quantified using a NanoDrop Spectrophotometer (Thermo Scientific). Purification of poly A^+^ RNA from total RNA was made using the Oligotex mRNA Midi Kit (Qiagen, Foster City, CA). PolyA^+^ RNA samples were used to first synthesizing the complementary DNA (cDNA) in the presence of amino allyl dUTP (aa-dUTP, Sigma-Aldrich, St. Louis, MO) and then chemically coupling the Cy3 or Cy5 dyes (Amersham-Pharmacia Biotech, Arlington Heights, IL). Briefly, 2 μg of each poly A^+^ RNA was annealed to 2 μl of random hexamer primers (Invitrogen, Carlsbad, CA, 3 mg/mL,) after denaturation at 70°C for 10 min. The synthesis of cDNA was allowed to proceed at 46°C for 4 hours in the presence of SuperScript II reverse transcriptase (400U per reaction; Invitrogen, Carlsbad, CA) and amino allyl-dNTP mix, in a 2:3 aa-dUTP:dTTP ratio. After RNA degradation and neutralization, unincorporated nucleotides were removed using QiaQuick PCR purification kit (Qiagen, Foster City, CA) with substitution of potassium phosphate buffers for the manufacturer’s wash and elution buffers, which contain free amines that may compete with the Cy dye coupling reaction. cDNA from each infected hamster was labeled with Cy3 and cDNA from each uninfected hamster labeled with Cy5 for 1 hour at room temperature in the dark. Unincorporated dyes were removed using QiaQuick PCR purification kit (Qiagen, Foster City, CA). Cy3- and Cy5- dye labeled probes (200 pmoles each) were combined and dried to completion. In general, labeled cDNA from 1 infected and 1 uninfected hamster were hybridized simultaneously onto a single microarray slide. In addition, one dye swap was made from each time point using pooled poly A^+^ RNA form each group so that each time point included a total of 6 microarray slides: 5 slides to which unique uninfected and infected samples were hybridized and 1 dye swap from pooled poly A^+^ RNA. Prior to hybridization, slides were rehydrated with warm 1X SSC buffer and snap dried for 5 seconds onto a preheated heat block at 110°C. To couple the DNA spots, slides were UV cross linked at 120 mJ and incubated in pre-hybridization buffer (5X SSC, 0.1% SDS and 1% BSA) at room temperature for 20 min followed by an additional incubation in pre-warm pre-hybridization buffer in a 42°C water bath for 45 minutes. Slides were washed with water, dipped in isopropanol and dried by centrifugation at 550 rpm for 5 minutes. Slides were hybridized within 1 hour of post-processing. Dry Cy3/Cy5 probe mixtures were resuspended in 24 μL of 1X hybridization buffer (50% formamide, 5X SSC and 0.1% SDS). To block nonspecific hybridization 20 μg of Mouse COT-1 DNA® (Invitrogen, Carlsbad, CA) and 20 μg of Poly (A)-DNA (Amersham-Pharmacia Biotech, Arlington Heights, IL) were added to each hybridization mixture. Post-processing slides were hybridized overnight at 42°C using hybridization chambers (Corning Costar, Acton, MA). After the hybridization step, slides were washed for 4 min at 42°C in low stringency buffer (1X SSC and 0.2% SDS), followed by two 2 min wash in a higher stringency buffer (0.1X SSC and 0.2% SDS) at room temperature, then by four 4 min wash in the highest stringency buffer (0.1X SSC) at room temperature and then dried by centrifugation at 550 rpm for 5 min. Hybridized slides were scanned at 635 nm and 532 nm on an Axon 4000 scanner (Axon Instruments, Foster City, Calif.). A more detailed protocol is available under request.

### Microarray data processing and analysis

Microarray slide data were quantified and .GPR files were created by using GenePix Pro version 3.0 software. Further analysis utilized Partek Genomics Suit (Partek GS), version 6.6, 2012 Partek Inc., St. Louis, MO, USA. Briefly, the GenePix .GPR files were imported into Partek GS, under the two-color microarray option. Channel 1 was assigned to the mean feature pixel intensity at 532 nm with the median background subtracted (F532 Mean - B532) and channel 2 was assigned to the mean feature pixel intensity at 635 nm with the median background subtracted (F635 Mean - 635). In general, data were imported only from un-flagged features and for gene IDs that appeared more than once in a file the mean was calculated and imported. The normalization step was performed using total intensity normalization and a log2 transformation to represent gene expression levels (for a review, see [67]). Each time point was analyzed separately and files within each time point were grouped into the uninfected and infected categories. Analysis of variance (ANOVA) was performed to generate the lists of genes differentially expressed between the uninfected and infected groups, and the batch effect ANOVA function of Partek was run to remove the effect of the dyes from the results. EST sets with a fold-change >2.0 or < −2 and adjusted p-value FDR <0.05 [68] were considered differentially expressed between the uninfected and infected groups.

### Gene symbol assignment

Column IDs from the lists of differentially expressed EST were loaded into the Hamster ESTIMA database to assign gene symbols of the orthologs of the closest match. When an ortholog gene symbol could not be assigned throughout the ESTIMA data base, the trimmed sequence of the unknown EST was compared to the nucleotide collection (nr/nt) using the following BLAST programs: Megablast, for comparing a query to closely related sequences, and Discontiguous megablast, for cross-species comparisons. Lists were manually curated to remove non-mammalian, short sequences, and sequences with no significant alignment to the to the nucleotide collection.

### Pathway analysis

The lists of differentially expressed genes generated by the microarray analysis were further analyzed using WebGestalt (http://bioinfo.vanderbilt.edu/webgestalt/; last updated on 1/30/2013) [33,34] and WikiPathways (http://www.wikipathways.org) [35] to identify signaling pathways significantly overrepresented in the infected spleen tissue.

### Quantitative RT-PCR

Total RNA was extracted from 20 μg of spleen tissue from uninfected hamsters or 28-day infected animals using the RNeasy kit (QIAGEN). All RNA samples were DNase treated with TURBO DNA-freeTM kit (Ambion®) and quantified using a NanoDrop Spectrophotometer (Thermo Scientific) and maintained at −80°C until ready to use. 250-500 ng of RNA was used for cDNA synthesis using the high capacity cDNA reverse transcription kit (Applied Biosystems). Gene expression was determined by SYBR green (Applied Biosystems) PCR using the following primers at a final concentration of 300–500 nM: CCL17: For–GTGCTGCCTGGAGATCTTCA, Rev–TGGCATCCCTGGGACACT; Arginase 1: For–ACCTATGTGTCATTTGGGTGGA, Rev–GCAGATATGCAGGGAGTCACC; CCL22: For–CGTGGCTCTCATCCTTCTTGC, Rev-CAGATGCTGTCTTCCACGTTGG; IDO: For–CACATGTCTCCCACTGAAGG, Rev-CAGGCACTGAATGTCTGAGG; IRG1: For–GAGAGGGTTGTGCTCAGGAT, Rev-CCACGTACTGGAAGGAGTGA; IFNγ: For–AATATCTTGACGAACTGGCAAA, Rev-CCTTCAAGGCTTCAAAGAGTTT; CXCL9: For–TGGGTATCATCCTCCTGGAC, Rev–AATGAGGACCTGGAGCAAAC; CXCL10: For–TGGAAATTATTCCTGCAAGTCA, Rev-GTGATCGGCTTCTCTCTGGT; CCL11: For–CTATCCCAGTTTCCTGCTGC, Rev-GGTCAGCACAGATATCCTTGC; IRF1: For–CAAGTCCAGCCGAGACACTA, Rev-GGTGTAGCTGCTGTGGTCAT; NOS2: For–TGAGCCACTGAGTTCTCCTAAGG, Rev-TCCTATTTCAACTCCAAGATGTTCTG; 18 s: For–ACCGCAGCTAGGAATAATGGA, Rev- GCCTCAGTTCCGAAAACCA. With the exception of CCL17 and IRG1 primers were designed to span an intron, and were confirmed by efficiency testing and analysis of dissociation curves to not generate primer dimers. Data was analyzed using the comparative Ct method, relative to uninfected control spleen, and with the 18S rRNA gene as the normalizer.

### Statistical analysis

Statistical analysis of the microarray data is described above. Differences in mRNA expression determined by qRT-PCR between non-infected and the 28-day infected animals were analyzed by two tail Man-Whitney test or two tail unpaired t-test using GraphPad Prism version 5.01 for Windows, GraphPad Software, San Diego California USA (www.graphpad.com).

### Availability of supporting data

The data sets supporting the results of this article are included within the article and its additional files:

## Additional files

Additional file 1: Table S1. Hamster EST similarities with sequences from other species. Additional file 2: Table S2. Differentially expressed genes in the spleen at 7 days post-infection. Additional file 3: Table S3. Differentially expressed genes in the spleen at 14 days post-infection. Additional file 4: Table S4. Differentially expressed genes in the spleen at 28 days post-infection. Additional file 5: Table S5. List of highly upregulated transcripts in hamster spleens at 28-days post-infection.

## Abbreviations

Arg1: Arginase 1
EST: Expressed sequence tag
VL: Visceral leishmaniasis
PCA: Principal component analysis
FDR: False discovery rate
ESTIMA: Expressed sequence tag information management and annotation
