# Supplementary material for: Transcriptional profiling of the spleen in progressive visceral leishmaniasis reveals mixed expression of type 1 and type 2 cytokine-responsive genes
Source: BMC Immunol. 2014 Nov 26;15:38. doi: 10.1186/s12865-014-0038-z (PMC4253007; doi:10.1186/s12865-014-0038-z)
Supplement: Additional file 1: Table S1. — Hamster EST similarities with sequences from other species. [file 12865_2014_38_MOESM1_ESM.docx]

**Table S1. Hamster EST similarities with sequences from other species**

| **Closest sequence match to non-hamster species** | **Number of ESTs** |
| --- | --- |
| Mouse | 2251 |
| Rat | 1410 |
| Human | 141 |
| Non-human primate | 86 |
| Other mammal | 78 |
| Non-mammalian | 191 |
| No match | 658 |
| Total | 5085 |
