# Supplementary material for: Transcriptional profiling of the spleen in progressive visceral leishmaniasis reveals mixed expression of type 1 and type 2 cytokine-responsive genes
Source: BMC Immunol. 2014 Nov 26;15:38. doi: 10.1186/s12865-014-0038-z (PMC4253007; doi:10.1186/s12865-014-0038-z)
Supplement: Additional file 5: Table S5. — List of highly upregulated transcripts in hamster spleens at 28-days post-infection. [file 12865_2014_38_MOESM5_ESM.docx]

**Table S5. List of highly upregulated transcripts in hamster spleens at 28-days post-infection.**

| **Gene ID** | **Fold-Change** | **Gene ID** | **Fold-Change** | **Gene ID** | **Fold-Change** | **Gene ID** | **Fold-Change** |
| --- | --- | --- | --- | --- | --- | --- | --- |
| *Bbs1* | 4629.03 | *Cyp2c29* | 373.218 | *F2* | 278.329 | *Tekt2* | 201.783 |
| *IFNG* | 1748.95 | *B4galt5* | 369.355 | *Crym* | 273.209 | *Cyp2b10* | 197.919 |
| *MCNDA* | 1285.99 | *Aldob* | 367.608 | *Wdr61* | 270.616 | *Chst4* | 197.327 |
| *Ccl11* | 1212.26 | *Cyp2c70* | 367.173 | *Zbtb8a* | 270.248 | *C1qtnf9* | 196.776 |
| *IL-23* | 890.156 | *Agpat2* | 366.164 | *RGD1308612* | 269.482 | *Nqo1* | 194.868 |
| *Itgax* | 823.063 | *Eif2c3* | 363.604 | *RRN3* | 265.956 | *Fbxo6* | 192.073 |
| *CCL20* | 749.904 | *Apoa5* | 363.029 | *Adh4* | 265.445 | *Ugt2b5* | 191.915 |
| *Batf2* | 724.559 | *Colec10* | 360.307 | *Steap1* | 260.576 | *Steap1* | 189.423 |
| *APOL9a* | 680.249 | *Cnot3* | 358.219 | *Abcb11* | 259.953 | *Cpm* | 186.153 |
| *IGHA1* | 653.512 | *Reg3g* | 348.906 | *Asgr1* | 258.882 | *Pdpn* | 185.355 |
| *Cxcl5* | 653.219 | *Apob* | 348.7 | *Cyp3a2* | 257.874 | *Acss3* | 183.834 |
| *Serpina3* | 599.494 | *Ass1* | 348.531 | *RGD1565421* | 257.803 | *Gjb3* | 180.702 |
| *IRF2* | 571.013 | *Slc28a3* | 347.261 | *Pzp* | 255.813 | *Mbl1* | 180.027 |
| *Fgg* | 562.034 | *Fam174a* | 347.053 | *Krt1* | 255.386 | *Asgr1* | 178.245 |
| *Alb* | 537.378 | *Stim2* | 346.89 | *Fga* | 252.547 | *Fah* | 176.279 |
| *Rc3h1* | 513.814 | *E130309D14Rik* | 346.584 | *St8sia6* | 251.646 | *Fgl1* | 176.163 |
| *Csf3* | 509.579 | *Gc* | 346.317 | *Map3k1* | 250.315 | *Gnmt* | 174.229 |
| *CYP2A9* | 497.806 | *Ftcd* | 344.596 | *Acsm2* | 248.633 | *Uox* | 172.905 |
| *Cxcl1* | 488.247 | *Gcgr* | 342.3 | *Krt5* | 245.22 | *F13b* | 172.388 |
| *Rbm16* | 481.519 | *9130016M20Rik* | 340.608 | *Mbl2* | 244.86 | *Sftpb* | 169.888 |
| *Chst12* | 458.716 | *AU021092* | 340.25 | *C9* | 244.14 | *Eya2* | 167.455 |
| *IRF1* | 439.994 | *Hsd3b1* | 339.181 | *Lipc* | 243.514 | *CAPZB* | 163.146 |
| *MTMR3* | 436 | *Rragb* | 337.948 | *Acmsd* | 241.435 | *Rdh1* | 162.065 |
| *Pah* | 435.997 | *Cyp2a3* | 336.449 | *Azgp1* | 234.928 | *Gnmt* | 161.198 |
| *Slco1a5* | 419.571 | *Trpc1* | 332.35 | *CYP4A2* | 232.884 | *Homez* | 155.624 |
| *Itih1* | 417.648 | *CYP4A2* | 331.639 | *Plunc* | 228.979 | *Timd2* | 155.049 |
| *Vom1r35* | 410.644 | *Dao* | 323.282 | *PID1* | 228.205 | *Serpina1b* | 151.187 |
| *Adhfe1* | 408.986 | *Apof* | 322.063 | *Pum1* | 227.541 | *Mug1* | 141.569 |
| *OVOS2* | 405.102 | *Clec10a* | 318.581 | *OTX2* | 226.917 | *Spp2* | 133.425 |
| *Akr1c2* | 405.024 | *Cfl2* | 317.914 | *Car3* | 226.295 | *Prr9* | 132.261 |
| *Hsd3b1* | 404.118 | *Ttr* | 313.929 | *Cndp1* | 226.206 | *Kcns1* | 126.949 |
| *Cyp2b10* | 395.738 | *Acsm2* | 313.729 | *Sult3a1* | 220.131 | *Ido1* | 107.032 |
| *Pde1c* | 394.052 | *Tmem116* | 312.433 | *Muc19* | 220.075 | *Ibsp* | 51.5955 |
| *Slc27a5* | 390.244 | *Abat* | 311.437 | *Cyp3a25* | 217.046 | *Cdo1* | 45.3927 |
| *Slc16a11* | 389.541 | *Tat* | 308.302 | *Ahctf1* | 213.506 | *Spp1* | 36.2551 |
| *Pggt1b* | 383.754 | *Rgn* | 305.455 | *Serpina6* | 210.896 | *Cxcl9* | 35.6402 |
| *Tbx3* | 382.919 | *Otc* | 296.857 | *Akr1c21* | 208.961 | *Irg1* | 29.1878 |
| *Krt7* | 382.725 | *Ube2g1* | 288.562 | *SRPX2* | 207.752 | *Clec6a* | 24.9502 |
| *Fabp7* | 382.06 | *AFF3* | 287.498 | *Rdh1* | 207.279 | *Cxcl9* | 22.3409 |
| *Cry1* | 377.083 | *Sftpd* | 287.333 | *Cyp3a7* | 206.495 |  |  |
| *Ttpa* | 375.347 | *Anxa13* | 280.365 | *Hpd* | 205.439 |  |  |
| *Rab11a* | 375.212 | *Hrg1* | 279.655 | *Krt23* | 203.478 |  |  |
